# Supplementary material for: Ultra-stable and highly reactive colloidal gold nanoparticle catalysts protected using multi-dentate metal oxide nanoclusters
Source: Nat Commun. 2024 Feb 6;15:851. doi: 10.1038/s41467-024-45066-9 (PMC10847421; doi:10.1038/s41467-024-45066-9)
Supplement: Supplementary file 1 — Supplementary Information [file 41467_2024_45066_MOESM1_ESM.pdf]

Supplementary Information

Ultra-stable and highly reactive colloidal gold nanoparticle catalysts protected using multi-dentate metal oxide nanoclusters

Kang Xia[1], Takafumi Yatabe[1], Kentaro Yonesato[1], Soichi Kikkawa[2], Seiji Yamazoe[2], Ayako Nakata[3], Ryo Ishikawa[4], Naoya Shibata[4], Yuichi Ikuhara[4], Kazuya Yamaguchi[1] & Kosuke Suzuki[1]*

[1]Department of Applied Chemistry, School of Engineering, The University of Tokyo, Tokyo, Japan

[2]Department of Chemistry, Graduate School of Science, Tokyo Metropolitan University, Tokyo, Japan

[3]International Center for Materials Nanoarchitectonics (WPI-MANA), National Institute for Materials Science (NIMS), Ibaraki, Japan

[4]Institute of Engineering Innovation, The University of Tokyo, Tokyo, Japan

*e-mail: ksuzuki@appchem.t.u-tokyo.ac.jp

Contents

	Page
1. Synthesis	2
2. Supplementary Figures	3–19
3. Supplementary Tables	20–22
4. Supplementary References	23–24

1. Synthesis

Preparation of supported gold nanoparticle catalysts

Gold nanoparticle catalyst supported on manganese oxide octahedral molecular sieve (Au/OMS-2) was prepared according to the reported procedure:[1] an aqueous solution of $HAuCl_4·4H_2O$ (8.3 mM, 60 mL) containing OMS-2 (2.0 g) was vigorously stirred at room temperature. After 15 min, the pH of the solution was quickly adjusted to 10 by addition of an aqueous solution of NaOH (1.0 M), and the resulting slurry was further stirred for 24 h. The solid was then filtered, and the residue was washed with a large amount of water (4 L), and dried in vacuo to afford the supported hydroxide catalyst precursor. Then, the hydroxide precursor was calcined at 300 °C for 2 h to give Au/OMS-2 as a dark brown powder (Au content: 3.6 wt%).

Gold nanoparticle catalyst supported on hydroxyapatite (Au/HAP) was prepared according to the reported procedure:[2] HAP (2.0 g) was added to an aqueous solution of $HAuCl_4$ (2 mM, 100 mL). After vigorously stirring the mixture for 2 min, aqueous NH_3 (10%, 240 μL) was added, and the resulting mixture was stirred at room temperature for 14 h. The resulting slurry was filtered, washed with deionised water (1 L) and dried at room temperature in vacuo to give the HAP-supported Au precursor. The resulting species was dispersed in deionised water (100 mL) and treated with $NaBH_4$ (80 mg) at room temperature for 1 h. The mixture was then filtered, and the residue was washed with water (1 L) and dried to afford Au/HAP as a reddish-purple powder (Au content as determined by ICP-AES: 1.5 wt%).

Preparation of TOASiW9 and TDASiW9 as reference materials

TOASiW9 and **TDASiW9** were prepared through phase transfer of **SiW9** using tetabutylammonium bromide (TOAB) and tetadecylammonium bromide (TDAB), respectively, as follows: an aqueous solution of **NaSiW9** (20 mL, 5 mM) was mixed with a solution of TOAB or TDAB in toluene (20 mL, 50 mM). The two-phase mixture was vigorously stirred for 30 min, followed by a phase-separation to yield the organic layer as the toluene solution of **TOASiW9** and **TDASiW9**.

2. Supplementary Figures

a

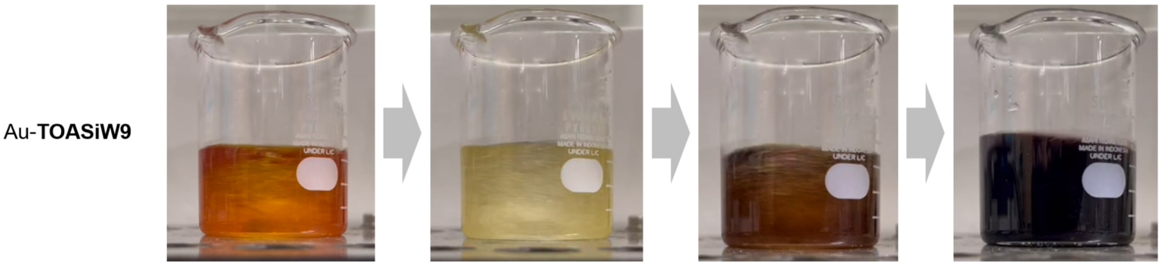

b

	Stage I	→ Stage II	→ Stage III	→ Stage IV
Time to spend * (Au-**TOASiW9**)	-	5 s	10 s	10 s
Time to spend * (Au-TOAB)	-	20 s	20 s	20 s
Possible Au species	Au^{3+}	Au^{3+}, Au^{+}	Au^{3+}, Au^{+}, Au^{0}	Au^{0}

* Time here refers to how long it costs till the next stage (or the point of color change)

Supplementary Fig. 1 | Synthesis of gold nanoparticles in this method: **a**, Photographs of reaction synthesis solutions of Au-**TOASiW9** in toluene after addition of the first drop of $NaBH_4$ as a reducing agent. **b**, Comparison of possible gold species in the reduction process of gold nanoparticles upon addition of $NaBH_4$ in the presence and absence of **SiW9** ligands.

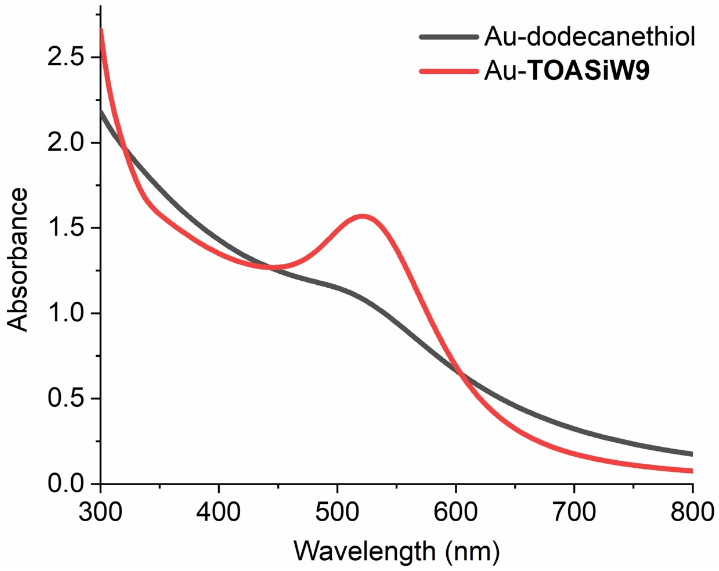

Supplementary Fig. 2 | UV-vis spectra of Au-**TOASiW9** and Au-dodecanethiol in toluene (Au 0.5 mM).

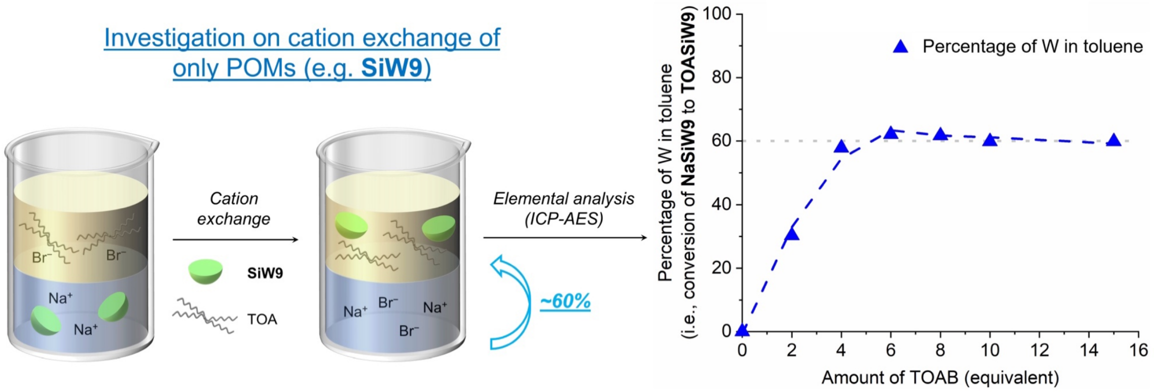

Supplementary Fig. 3 | Results of elemental analysis on the toluene phase after the transfer of **SiW9** from aqueous phase (**NaSiW9**) to toluene phase (**TOASiW9**) using various amount of TOAB as a phase transfer agent.

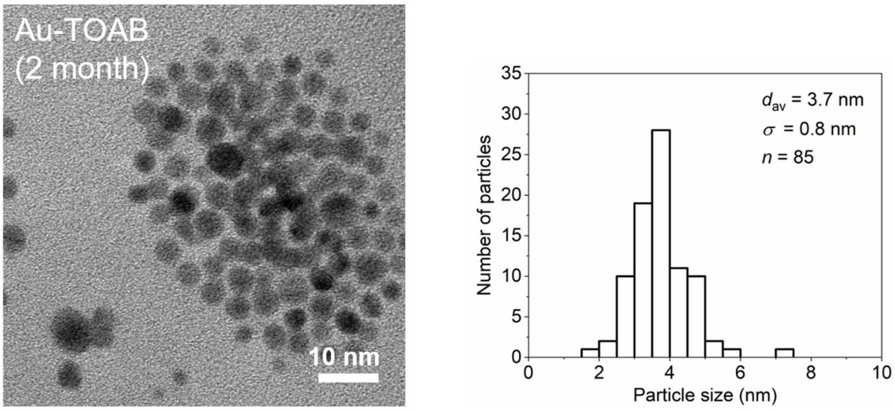

Supplementary Fig. 4 | TEM images and corresponding size distribution histograms of Au-TOAB after storage for 2 months.

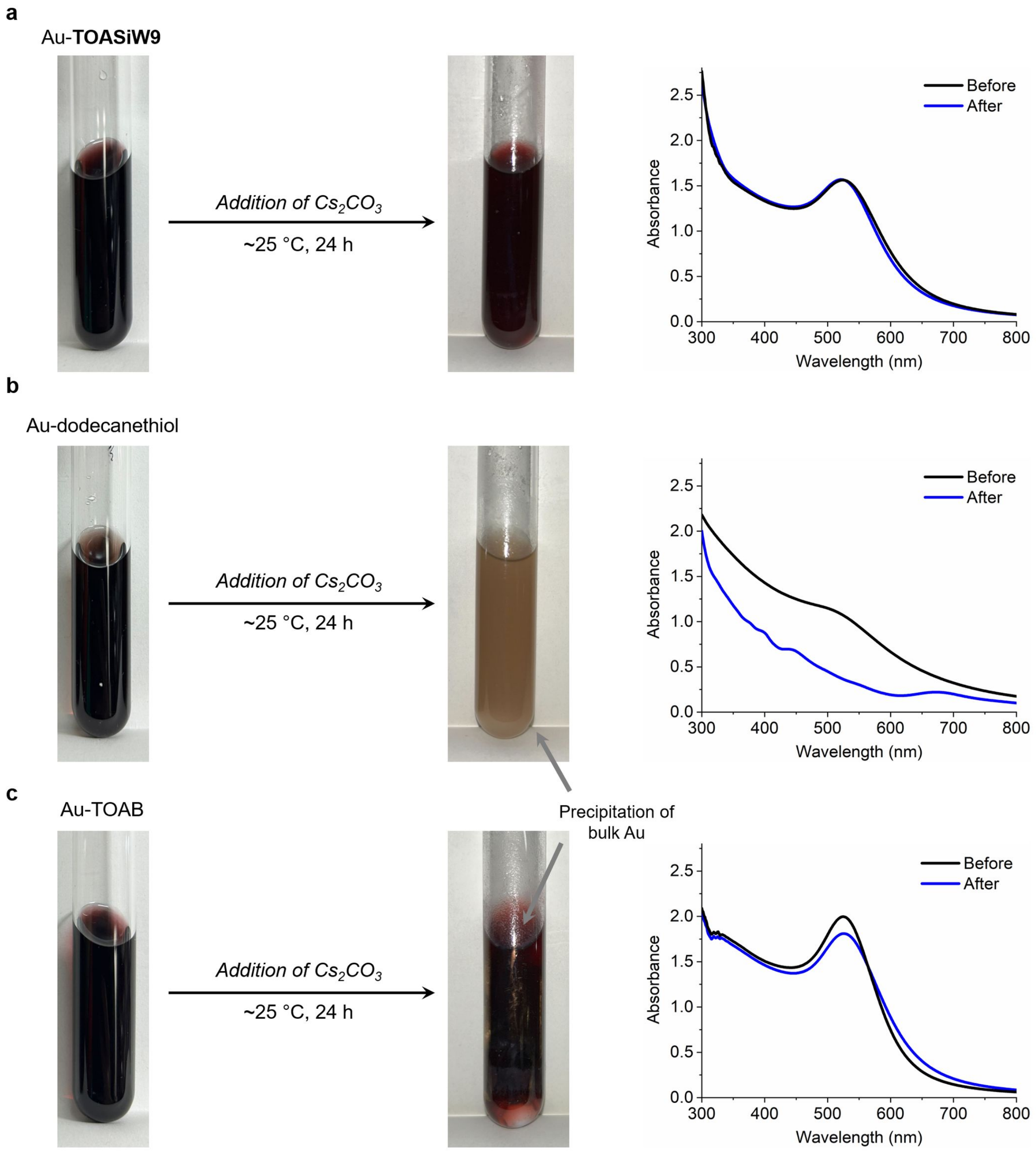

Supplementary Fig. 5 | Photographs and UV–vis spectra of the solutions of various ligand-protected gold nanoparticles in toluene before and after the addition of Cs_2CO_3 and stirring at room temperature for 24 h: **a**, Au-**TOASiW9**, **b**, Au-dodecanethiol, **c**, Au-TOAB. Au-dodecanethiol and Au-TOAB agglomerated and partially precipitated. Reaction conditions: 3 mL toluene solution of colloidal gold nanoparticles (Au: 0.01 mmol),Cs_2CO_3 (0.5 mmol), room temperature (~25 °C), O_2 (1 atm), 24 h.

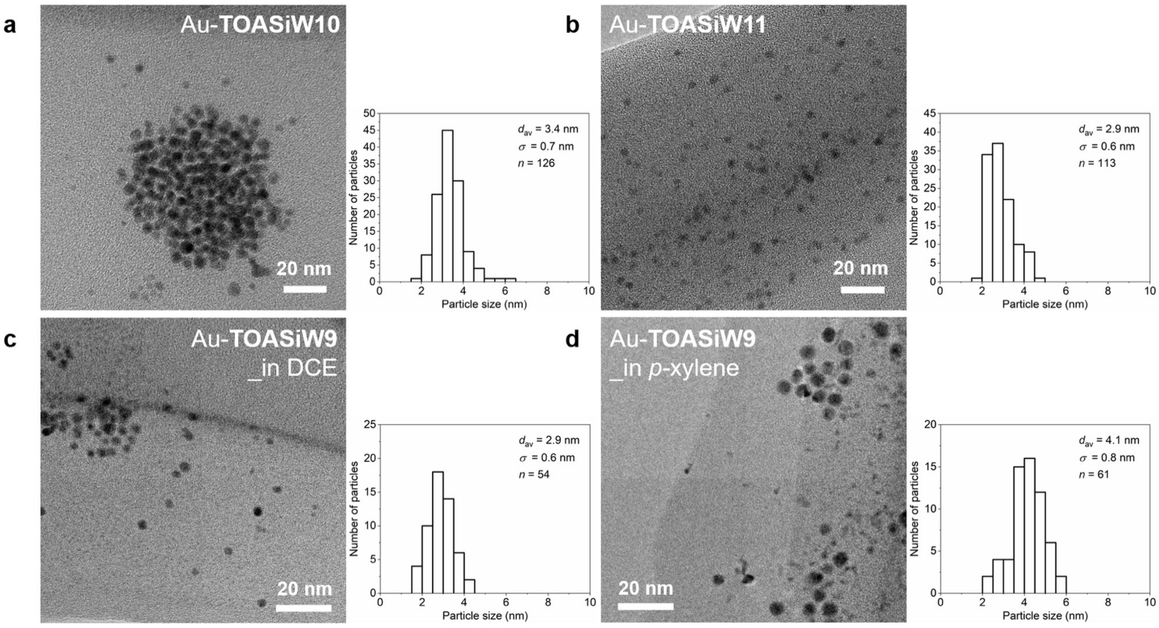

Supplementary Fig. 6 | TEM images and corresponding histograms of size distribution of **a**, Au-**TOASiW10** in toluene, **b**, Au-**TOASiW11** in toluene, **c**, Au-**TOASiW9** in dichloroethane (DCE) and **d**, Au-**TOASiW9** in *p*-xylene.

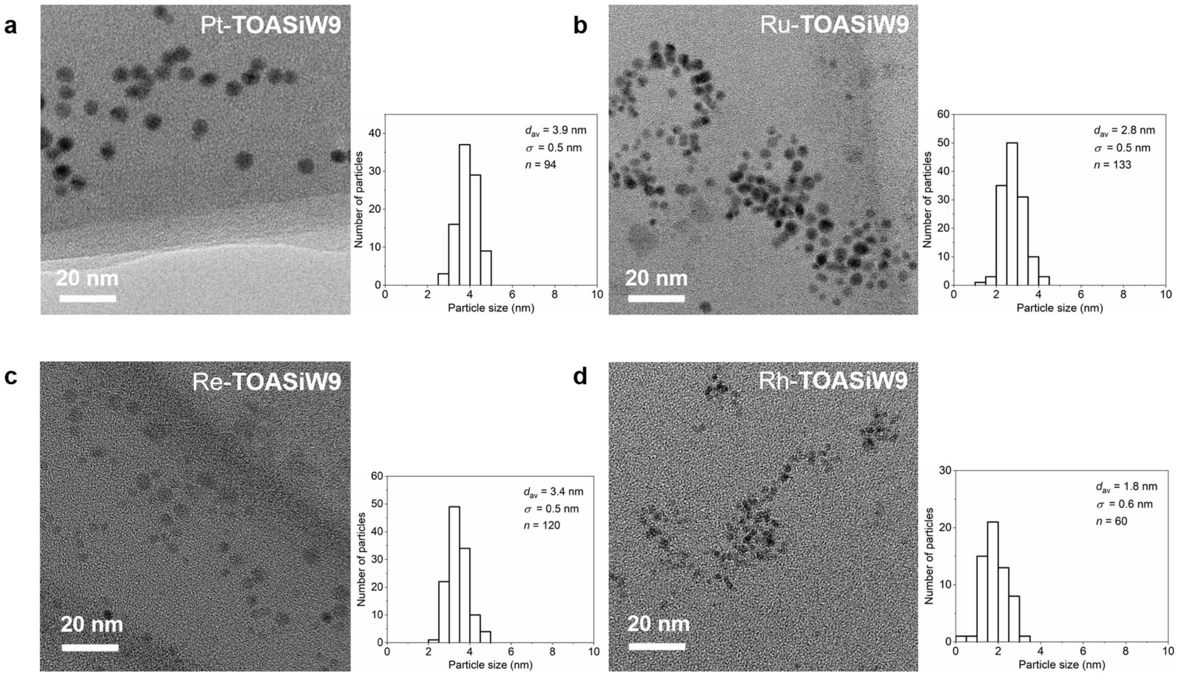

Supplementary Fig. 7 | TEM images and corresponding size distribution histograms of **TOASiW9**-protected metal nanoparticles prepared in toluene: **a**, platinum **b**, ruthenium **c**, rhenium and **d**, rhodium nanoparticles.

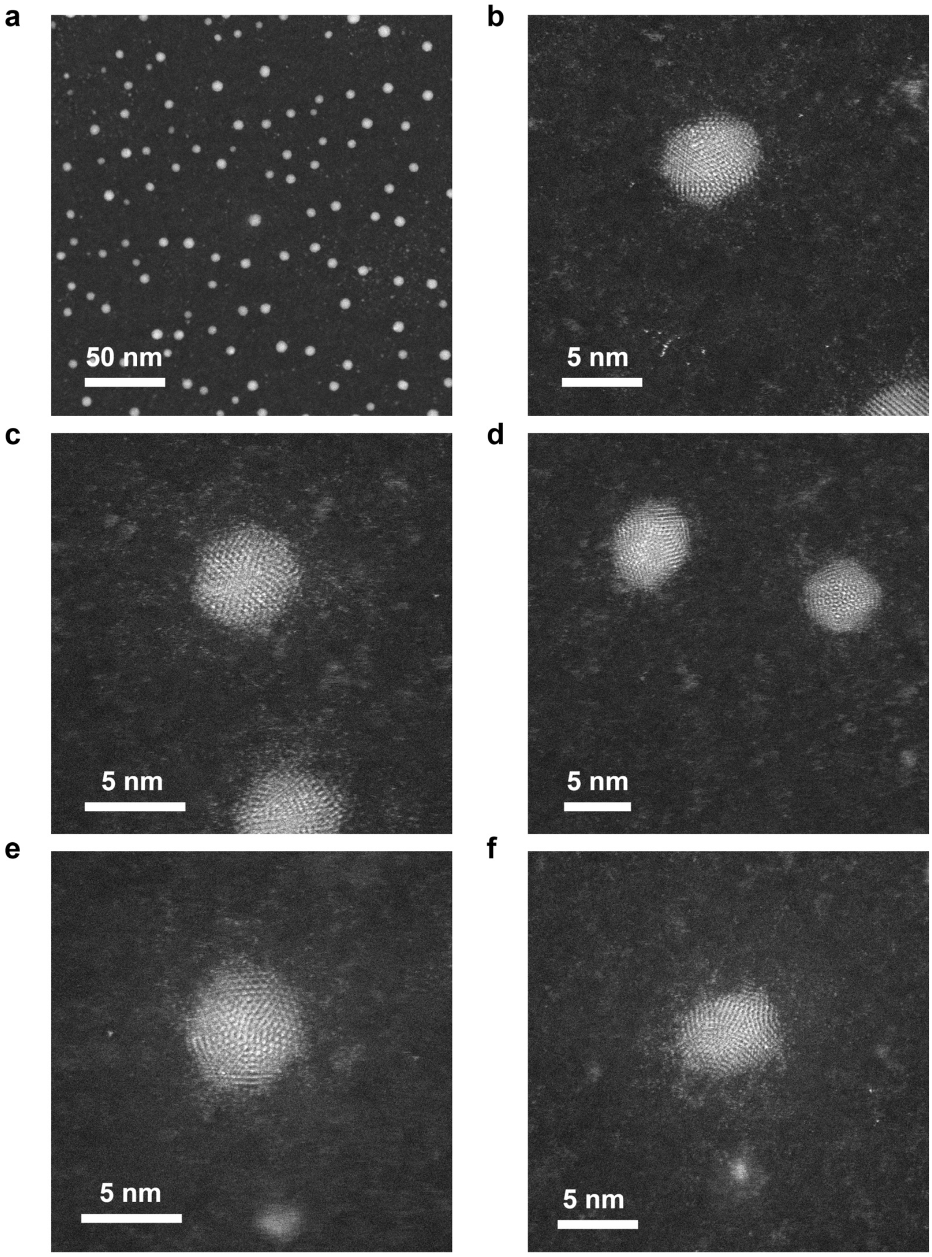

Supplementary Fig. 8 | Several selected ADF-STEM images of Au-**TOASiW9**. **a**, Low-magnification and **b-f**, high-magnification images.

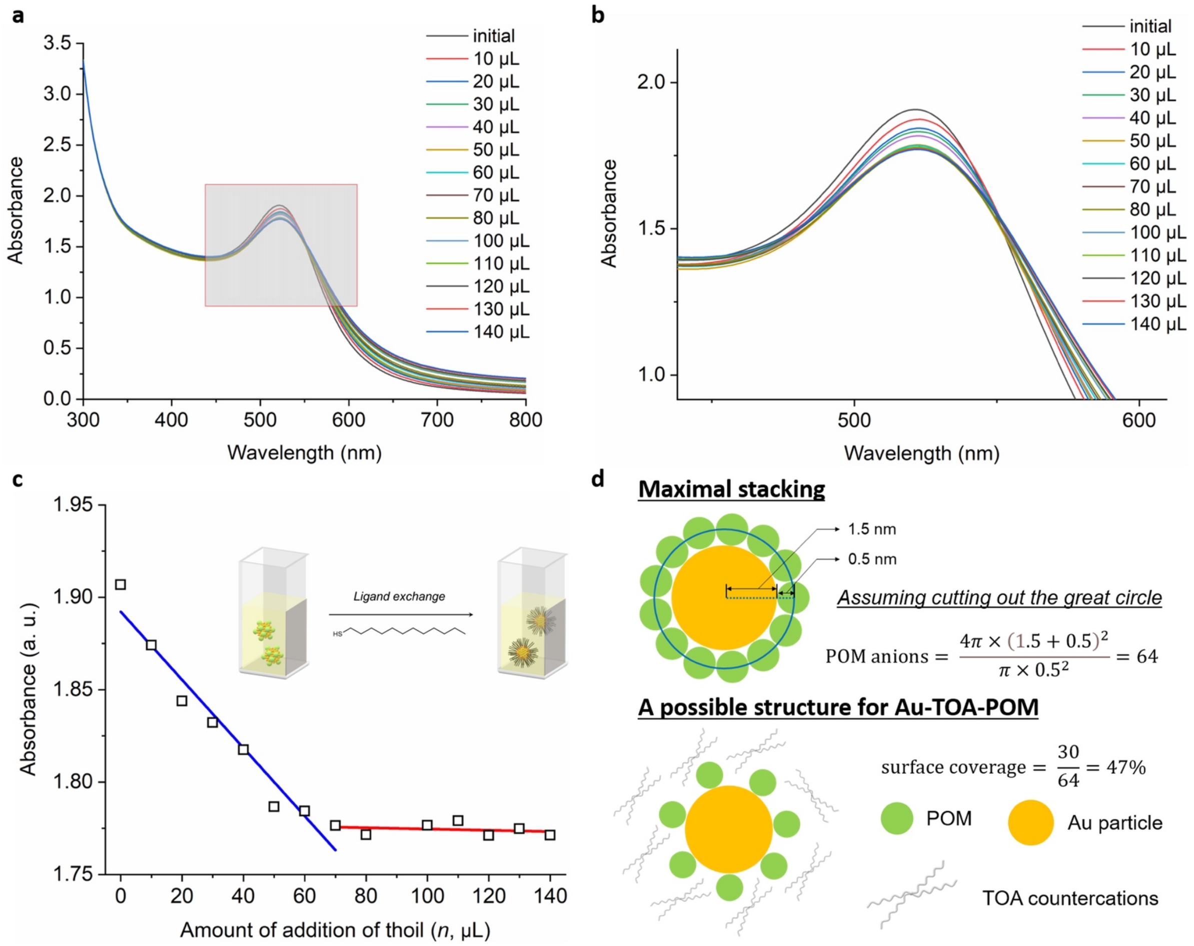

Supplementary Fig. 9 | Titration experiments using dodecanethiol in confirming surface coverage of Au-**TOASiW9**: **a**,**b**, UV-vis spectra of Au-**TOASiW9** (0.5 mM) in toluene upon addition of toluene solution of dodecanethiol (5 mM). **c**, A plot showing the change of the absorbance of SPR band (524 nm) upon addition of a toluene solution of dodecanethiol. **d**, Illustration for Au-**TOASiW9**.

The UV-vis spectra exhibit the continuous decrease of SPR band with showing isosbestic points upon addition of toluene solution of dodecanethiol, and then become constant after addition of 70 μL of the solution (dodecanethiol concentration, 1.56×10^{-4} mol/L). Based on the differences in refractive indices (*J. Phys. Chem. B* **2005**, *109*, 21556−21565; *Chem. Rev.* **2008**, *108*, 462−493), these results indicate the ligand exchange from POMs to dodecanethiols. Based on the density and molar mass of Au (19.3 g/cm^3 and 197 g/mol), assuming a spherical shape and a uniform face-centered cubic structure, the average number of gold atoms in 3 nm gold nanoparticle is calculated as $N = \frac{\rho V}{M} \times N_A = \frac{\pi(3\times10^{-7})^3}{6} \times \frac{19.3}{197} \times 6.02 \times 10^{23} = 834$. Based on total concentration of gold atoms (from chloroauric acid as 5×10^{-4} M, gold nanoparticle concentration is 6×10^{-7} mol/L. Thus, the number of dodecanethiol molecules in one gold nanoparticle is calculated as $\frac{1.56\times10^{-4}}{6\times10^{-7}} = 260$. Based on previous knowledges that an average ratio as 8.5 during the ligand exchange process from mono-lacunary POMs ($[AlW_{11}O_{39}]^{9-}$) to 11-mercaptoundecanoate (*J. Am. Chem. Soc.* **2009**, *131*, 17412−17422; *ACS Nano* **2012**, *6*, 629−640), there should be around 30 POM ligands surrounding a 3 nm gold particle in this case, and surface coverage ratio can be also estimated as 47%.

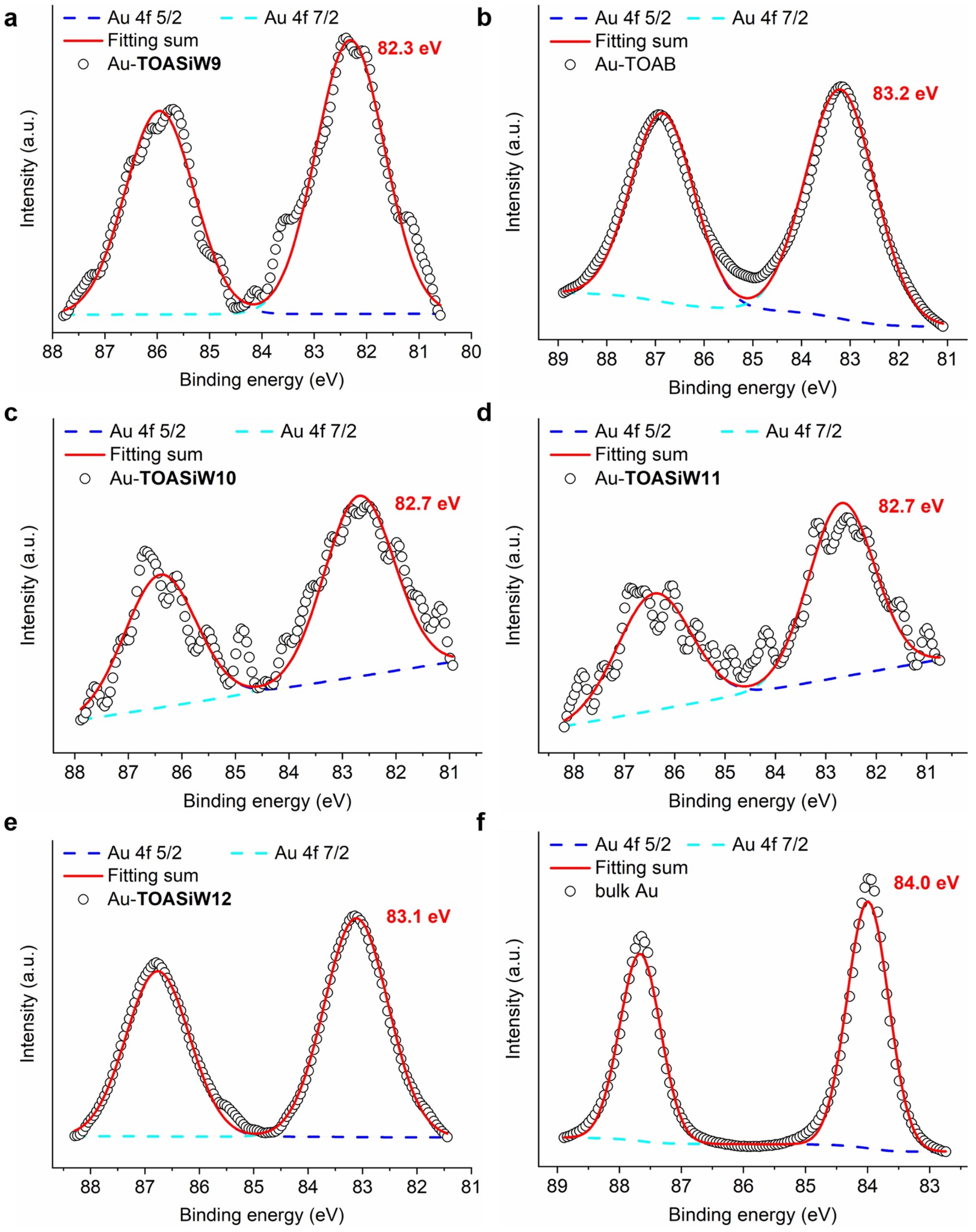

Supplementary Fig. 10 | XPS spectra of various ligand-protected gold nanoparticles: **a**, Au-**TOASiW9**. **b**, Au-TOAB. **c**, Au-**TOASiW10**. **d**, Au-**TOASiW11**. **e**, Au-**TOASiW12**. **f**, bulk Au (CAS No. 7440-57-5).

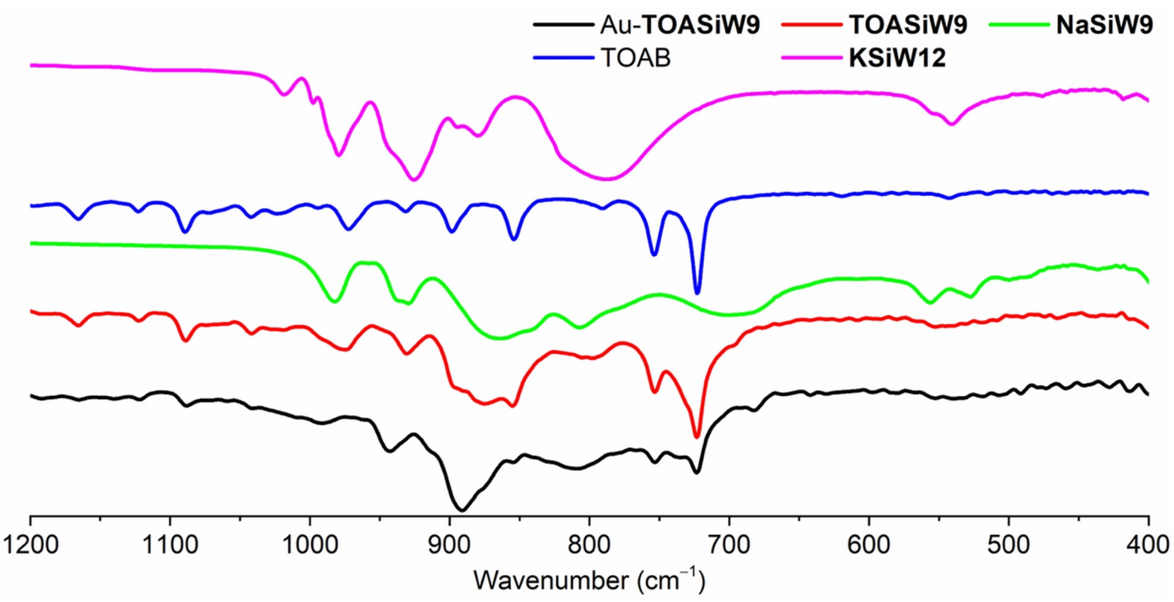

Supplementary Fig. 11 | IR spectra of Au-**TOASiW9**, **TOASiW9**, **NaSiW9**, TOAB and **KSiW12**.

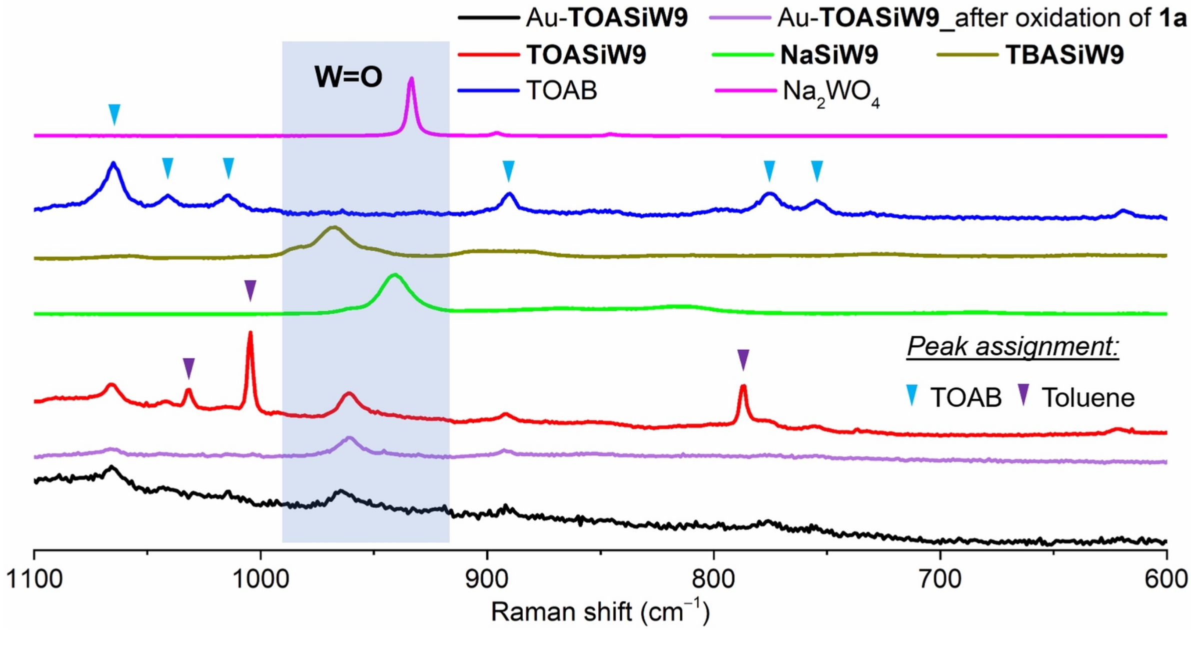

Supplementary Fig. 12 | Raman spectra of Au-**TOASiW9**, Au-**TOASiW9** after the catalytic oxidation of **1a**, **TOASiW9**, **NaSiW9**, **TBASiW9**, TOAB and sodium tungstate (Na_2WO_4).

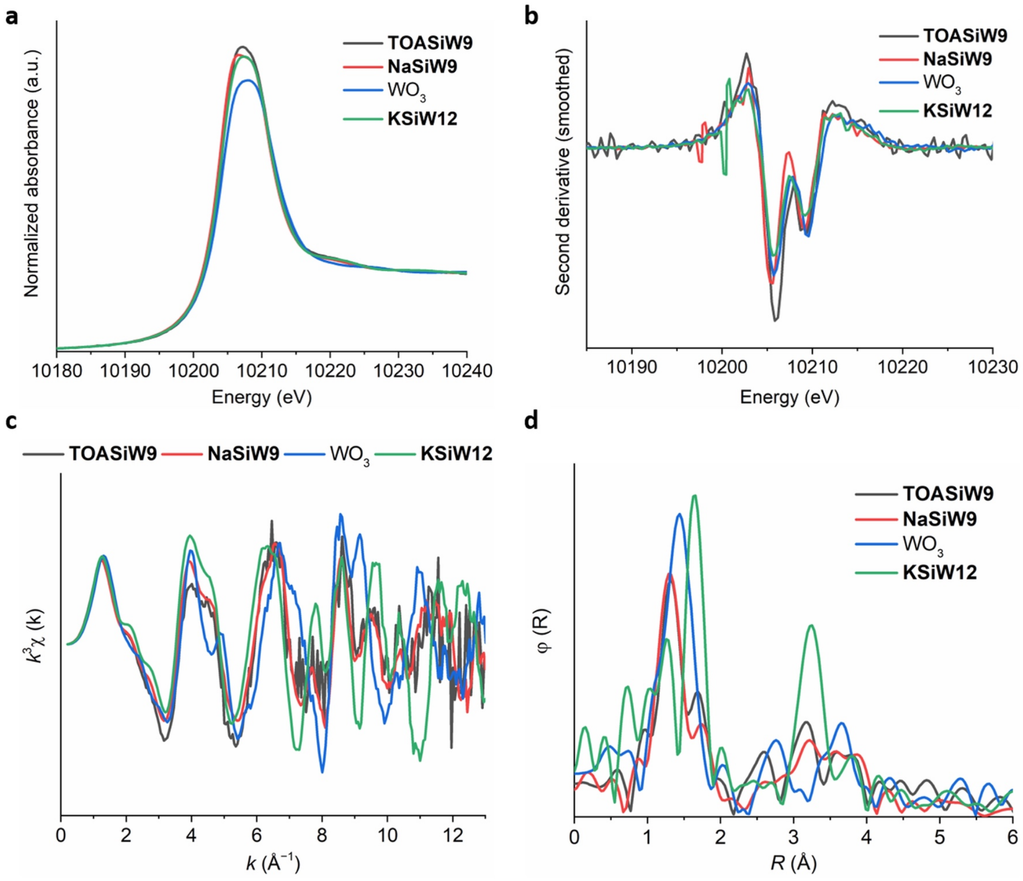

Supplementary Fig. 13 | XAFS studies of **TOASiW9**, **NsSiW9**, WO_3 and **KSiW12**: **a**, W-L_3-edge XANES spectra in wide range. **b**, Second derivatives in white line area. **c**, k^3-Weighted W L_3-edge EXAFS spectra. **d**, Fourier-transformed *R*-space EXAFS spectra.

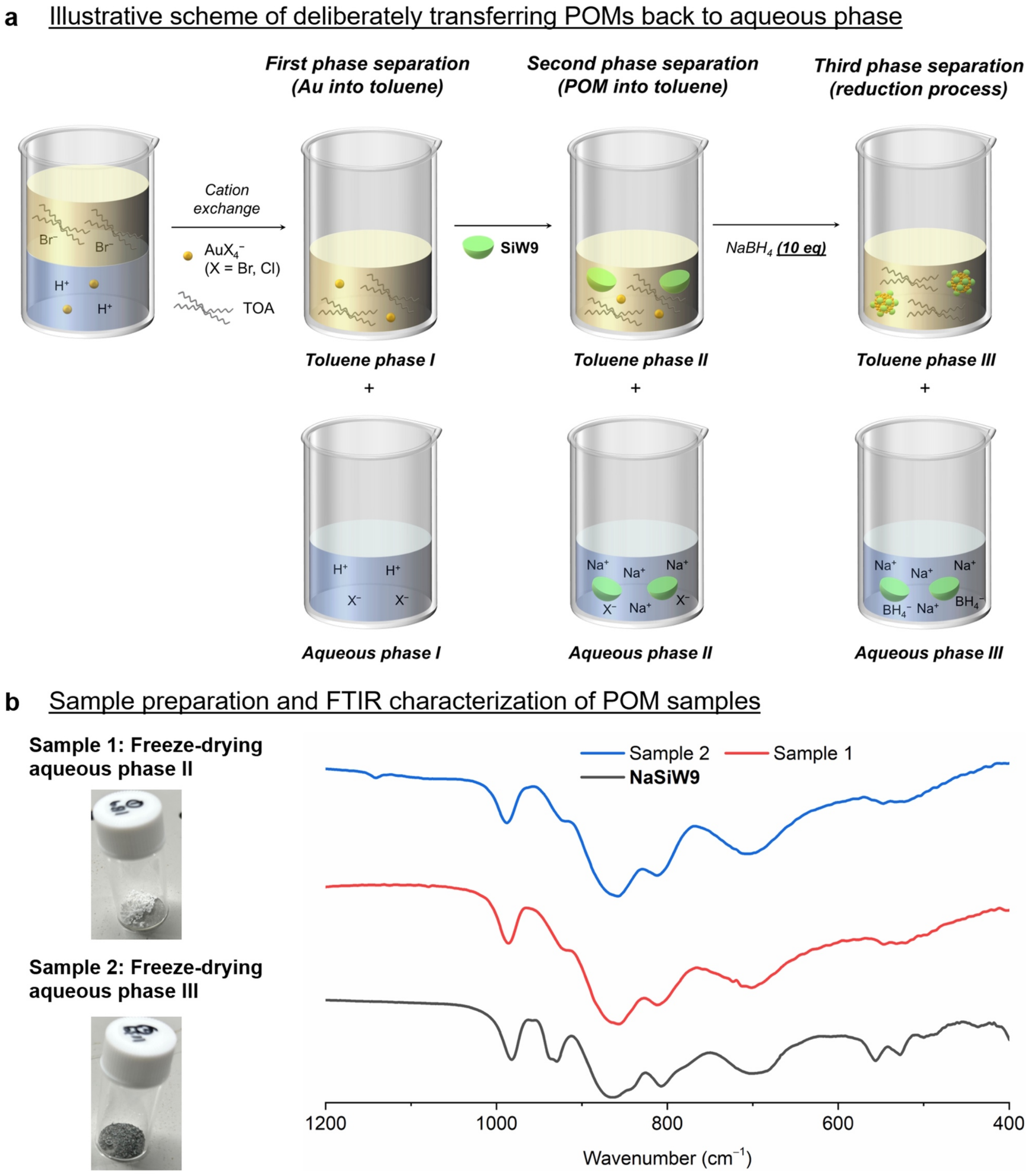

Supplementary Fig. 14 | Confirmation of structures of POMs in aqueous phase during synthesis after mixing with gold precursors and addition of sodium borohydride. **a**, Illustrative scheme of operative procedure. **b**, Photographs of solid samples by freeze-drying aqueous phase containing POMs and their IR spectra in comparison to **NaSiW9**.

During the optimization of amount of TOAB and $NaBH_4$ (Table S1), most of POMs in toluene phase were found to be reversed back to aqueous phase according the elemental analysis results (Table S1, Entry 5). Unsurprisingly, as-obtained gold nanoparticles agglomerated and aggregated within one month; whereas, this "undesired" finding can be utilized here to confirm POM structures during synthesis. In the IR spectra, the characteristic peaks of **SiW9** in the region of 500 – 1000 cm^{-1} were well consistent between **NaSiW9** and POMs after mixing with gold precursors and sodium borohydride respectively, indicating their intact structures in this method.

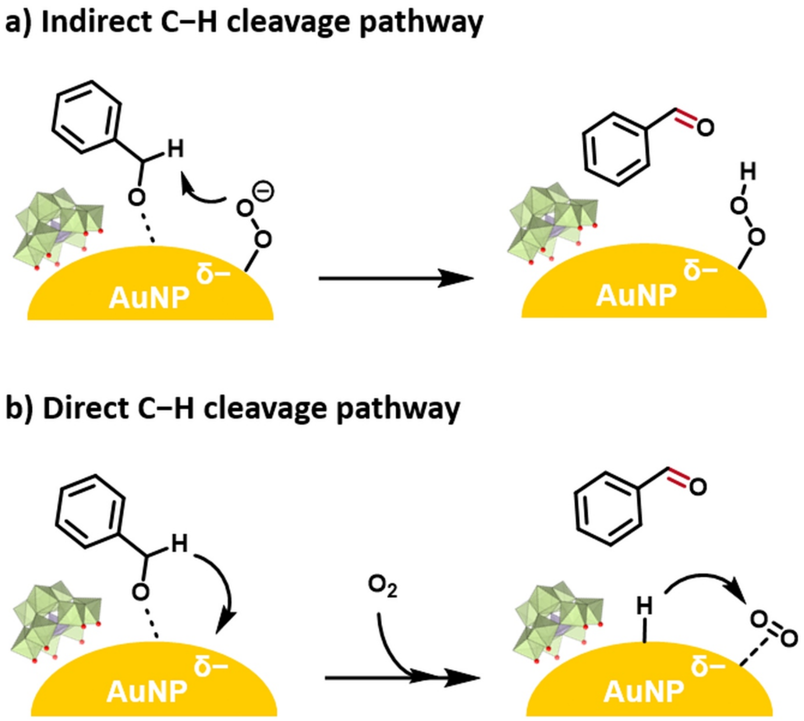

Supplementary Fig. 15 | Possible routes of Au-**TOASiW9**-catalysed aerobic oxidation of **1a**: **a**, Indirect C–H cleavage pathway. **b**, Direct C–H cleavage pathway. In both possible reaction pathways, efficient activation of O_2 can be considered to facilitate the aerobic oxidation.

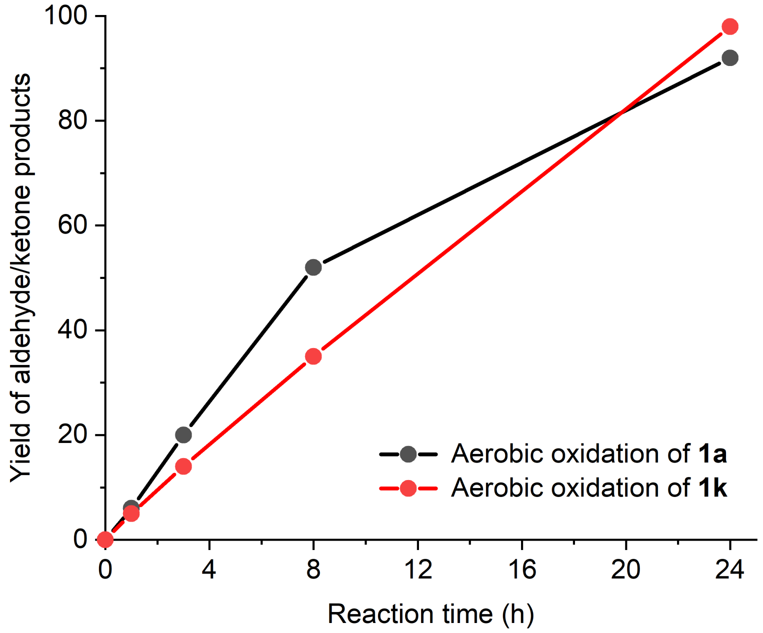

Supplementary Fig. 16 | Reaction profiles of catalytic oxidation of **1a** and **1k**. Reaction conditions: **1a** or **1k** (0.25 mmol), 3 mL toluene solution of colloidal gold nanoparticles (Au: 4 mol%), Cs_2CO_3 (0.5 mmol), room temperature (~25 °C), O_2 (1 atm), 24 h. All the reaction yields were determined via GC analysis using biphenyl as an internal standard.

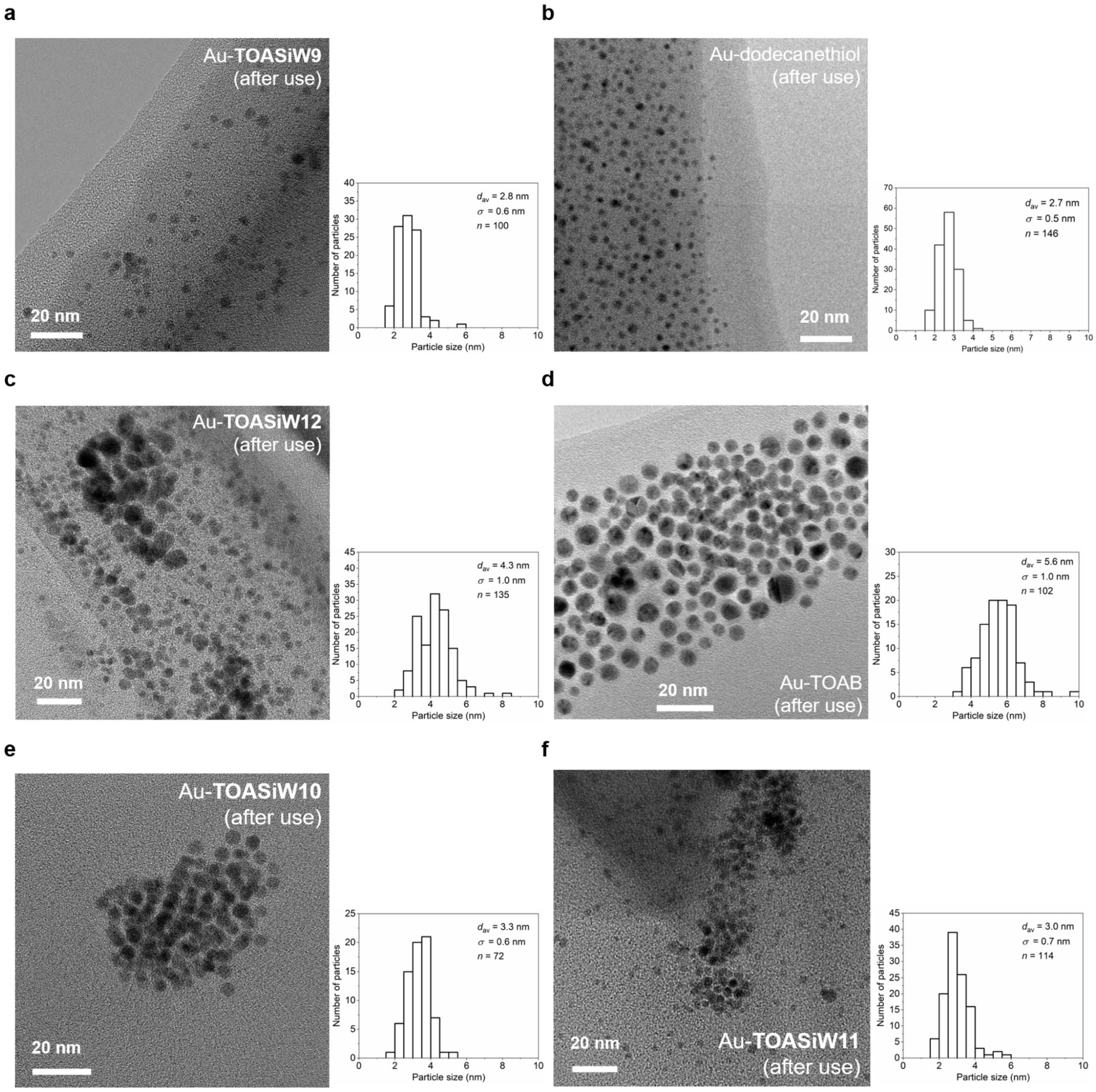

Supplementary Fig. 17 | TEM images and size distribution histograms of gold nanoparticles protected by various ligands after the catalytic alcohol oxidation: **a**, Au-**TOASiW9**. **b**, Au-dodecanethiol. **c**, Au-**TOASiW12**. **d**, Au-TOAB. **e**, Au-**TOASiW10**. **f**, Au-**TOASiW11**. Reaction conditions: **1a** (0.25 mmol), K_2CO_3 (0.5 mmol), catalyst (Au: 4 mol%), toluene (3 mL), room temperature (~25 °C), 24 h, O_2 (1 atm).

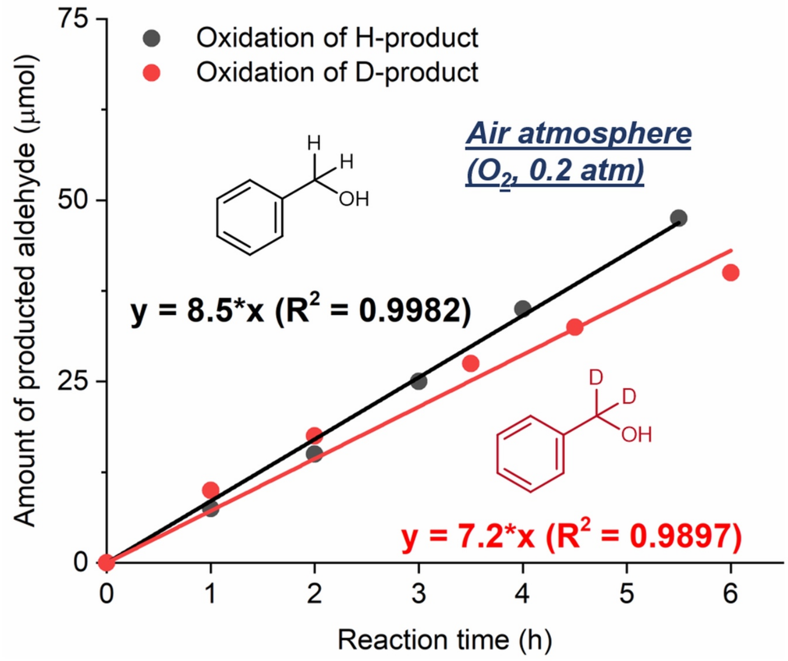

Supplementary Fig. 18 | Kinetic isotope effect test for the aerobic oxidation reaction of benzyl alcohol under O_2 (1 atm). Reaction conditions: benzyl alcohol or benzyl-α,α-d_2 alcohol (0.25 mmol), 3 mL toluene solution of Au-**TOASiW9** (Au: 4 mol%), biphenyl (0.1 mmol, internal standard), Cs_2CO_3 (0.5 mmol), room temperature (~25 °C), O_2 (1 atm).

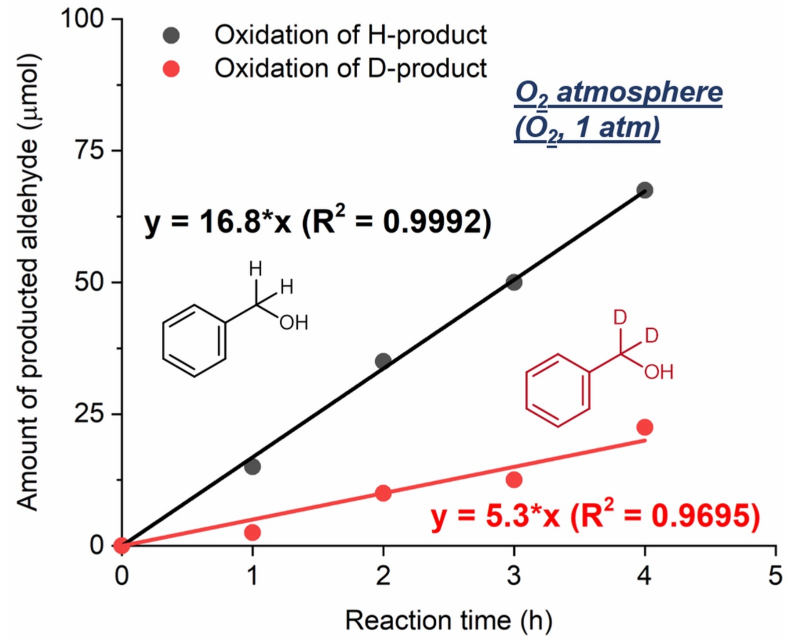

Supplementary Fig. 19 | Kinetic isotope effect test for the aerobic oxidation reaction of benzyl alcohol under air. Reaction conditions: benzyl alcohol or benzyl-α,α-d_2 alcohol (0.25 mmol), 3 mL toluene solution of Au-**TOASiW9** (Au: 4 mol%), biphenyl (0.1 mmol, internal standard), Cs_2CO_3 (0.5 mmol), room temperature (~25 °C), open air.

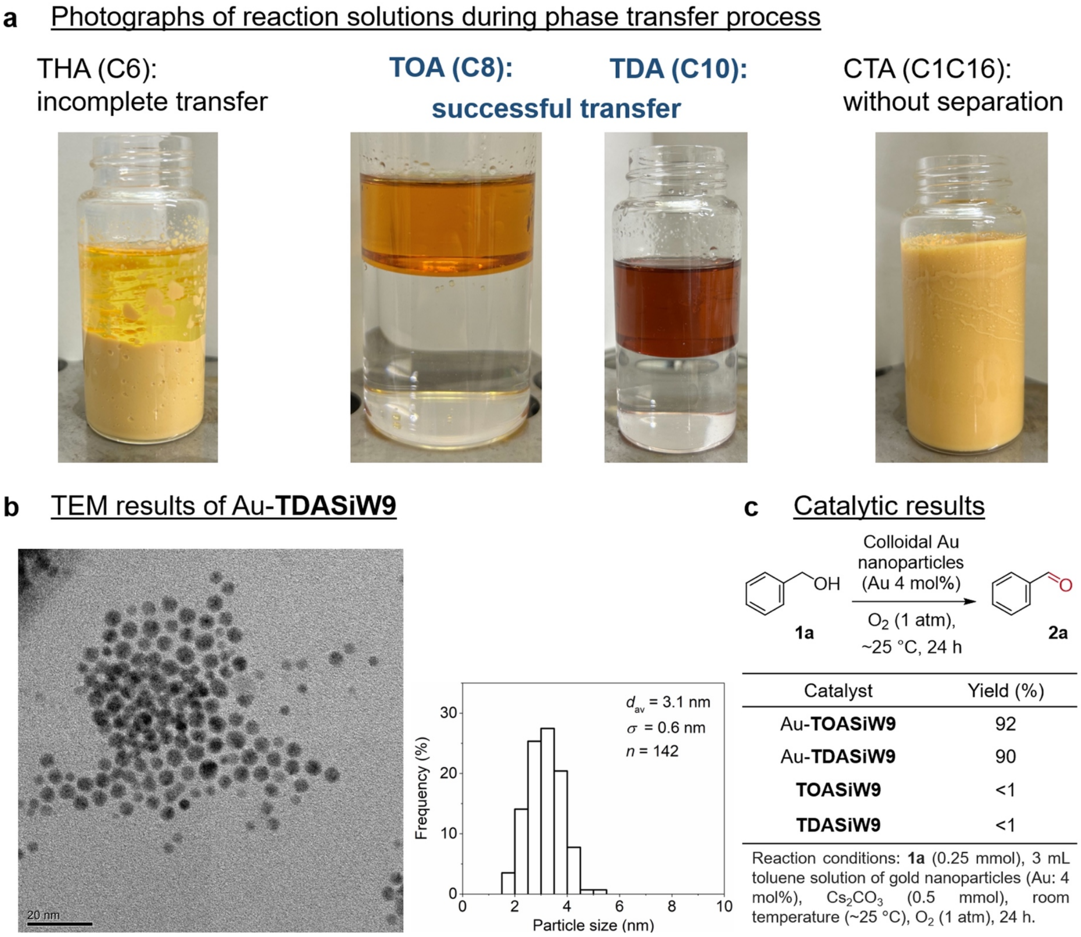

Supplementary Fig. 20 | Investigation on the effect of cations during phase transfer process of chloroauric acid into toluene phase with different surfactants. **a**, Photographs of reaction solutions during phase transfer process (top phase, toluene; bottom phase, H_2O). **b**, TEM image and size distribution histogram of Au-**TDASiW9**. **c**, The results of catalytic aerobic oxidation of **1a**.

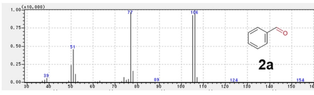

2a

CAS No. 100-52-7, MS (EI): *m/z* (%): 107 (8), 106 (100) [M^+], 105 (93), 78 (16), 77 (97), 74 (8), 52 (12), 51 (46), 50 (24)

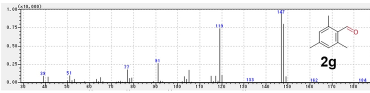

2g

CAS No. 487-68-3, MS (EI): *m/z* (%): 148 (81) [M^+], 147 (100), 120 (11), 119 (75), 115 (9), 105 (17), 91 (26), 77 (18), 51 (10), 39 (10)

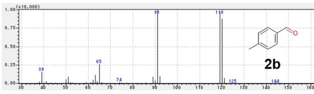

2b

CAS No. 104-87-0, MS (EI): *m/z* (%): 120 (88) [M^+], 119 (97), 92 (11), 91 (100), 89 (9), 65 (27), 63 (12), 39 (16), 51 (9)

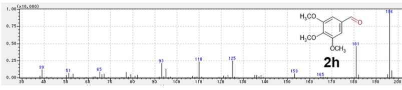

2h

CAS No. 86-81-7, MS (EI): *m/z* (%): 197 (11), 196 (100) [M^+], 181 (46), 125 (25), 110 (24), 95 (13), 93 (21), 39 (13)

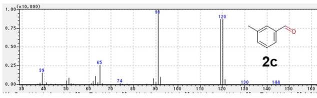

2c

CAS No. 620-23-5, MS (EI): *m/z* (%): 120 (88) [M^+], 119 (87), 92 (11), 91 (100), 89 (9), 65 (26), 63 (12), 39 (16), 51 (9)

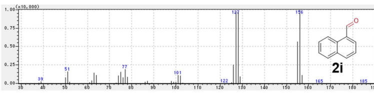

2i

CAS No. 66-77-3, MS (EI): *m/z* (%): 156 (100) [M^+], 155 (57), 128 (98), 127 (98), 126 (26), 77 (19), 75 (16), 51 (17)

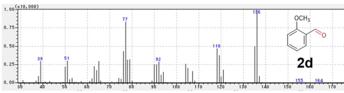

2d

CAS No. 135-02-4, MS (EI): *m/z* (%): 136 (100) [M^+], 135 (50), 119 (38), 118 (47), 92 (29), 79 (32), 78 (32), 77 (84), 76 (44), 65 (30), 51 (30), 39 (30)

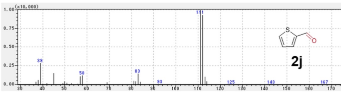

2j

CAS No. 98-03-3, MS (EI): *m/z* (%): 112 (93) [M^+], 111 (100), 83 (15), 58 (12), 57 (11), 45 (15), 39 (29)

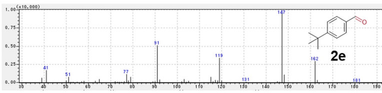

2e

CAS No. 939-97-9, MS (EI): *m/z* (%): 162 (30) [M^+], 148 (11), 147 (100), 119 (34), 115 (8), 91 (52), 79 (8), 77 (12), 41 (17)

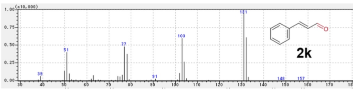

2k

CAS No. 14371-10-9, MS (EI): *m/z* (%): 132 (61) [M^+], 131 (100), 104 (27), 103 (60), 78 (38), 77 (48), 51 (41), 50 (14)

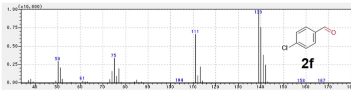

2f

CAS No. 104-88-1, MS (EI): *m/z* (%): 142 (25), 141 (38), 140 (76) [M^+], 139 (100), 113 (22), 111 (67), 77 (20), 75 (34), 51 (20), 50 (30)

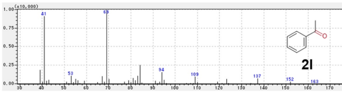

2l

CAS No. 98-86-2, MS (EI): *m/z* (%): 137 (8), 123 (7), 120 (1) [M^+],119 (3), 109 (10), 94 (16), 84 (25), 83 (11), 69 (100), 67 (11), 53 (11), 41 (92), 39 (19)

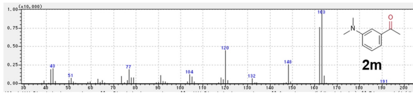

2m

CAS No. 18992-80-8, MS (EI): *m/z* (%): 164 (11), 163 (100) [M^+], 162 (88), 148 (26), 120 (46), 77 (20), 43 (21), 42 (19)

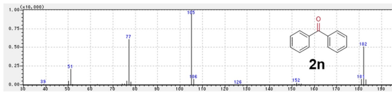

2n

CAS No. 119-61-9, MS (EI): *m/z* (%): 183 (7), 182 (52) [M^+], 181 (8), 106 (8), 105 (100), 77 (62), 76 (6), 51 (22), 50 (6)

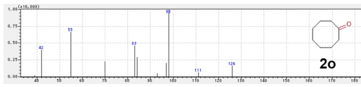

2o

CAS No. 502-49-8, MS (EI): *m/z* (%): 126 (17) [M^+], 111 (7), 98 (100), 97 (21), 84 (30), 83 (46), 70 (23), 55 (67), 42 (40)

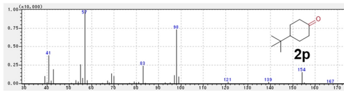

2p

CAS No. 98-53-3, MS (EI): *m/z* (%): 154 (16) [M^+], 98 (73), 97 (12), 83 (24), 69 (14), 57 (100), 55 (26), 43 (19), 41 (39), 39 (10)

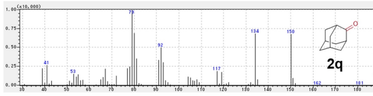

2q

CAS No. 700-58-3, MS (EI): *m/z* (%): 150 (68) [M^+], 134 (69), 93 (31), 92 (50), 91 (33), 81 (36), 80 (69), 79 (100), 41 (27)

Supplementary Fig. 21 | MS (EI) spectra of aldehyde/ketone products.

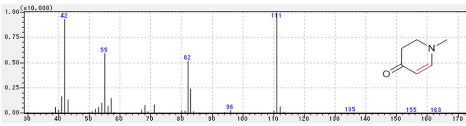

CAS No. 35488-00-7, MS (EI): *m/z* (%): 111 (100) [*M*+], 83 (24), 82 (52), 57 (15), 55 (59), 43 (13), 42 (94), 41 (17)

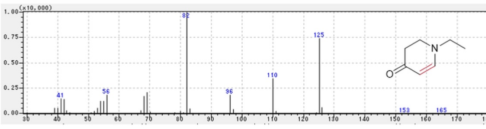

CAS No. 55176-40-4, MS (EI): *m/z* (%): 125 (74) [*M*+], 110 (35), 96 (18), 82 (100), 69 (21), 68 (17), 56 (19)

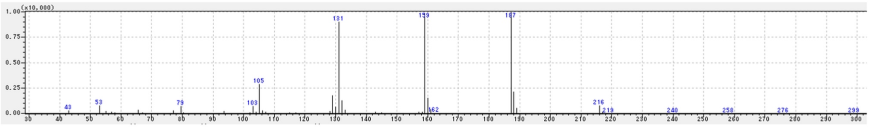

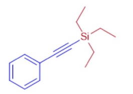

CAS No. 4131-43-5, MS (EI): *m/z* (%): 216 (8) [*M*+], 188 (22), 187 (100), 160 (16), 159 (99), 132 (13), 131 (91), 129 (18), 105 (29)

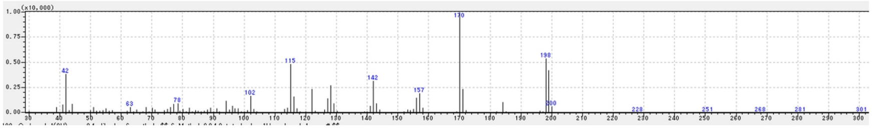

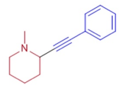

CAS No. 51498-55-6, MS (EI): *m/z* (%): 199 (42) [*M*+], 198 (54), 170 (100), 142 (31), 128 (27), 122 (24), 115 (49), 42 (38)

Supplementary Fig. 22 | MS (EI) spectra of products in reaction scope.

(a) Oxidative dehydrogenation of N-alkyl-4-piperidone to corresponding enaminone products

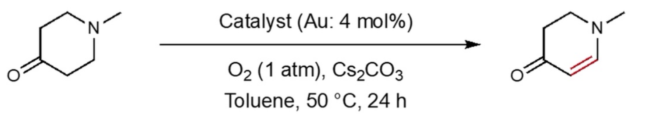

Catalyst	Yield (%)
Au-**TOASiW9**	**41**
Au/OMS-2	1
Au/HAP	<1
Au-**TOASiW9** (Au: 10 mol%, 70 °C)	**79**

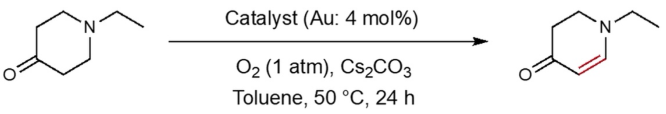

Catalyst	Yield (%)
Au-**TOASiW9**	**32**
Au/OMS-2	<1
Au/HAP	<1
Au-**TOASiW9** (Au: 10 mol%, 70 °C)	**55**

(b) Cross-dehydrogenative coupling (CDC) of ethynylbenzene and triethylsilane

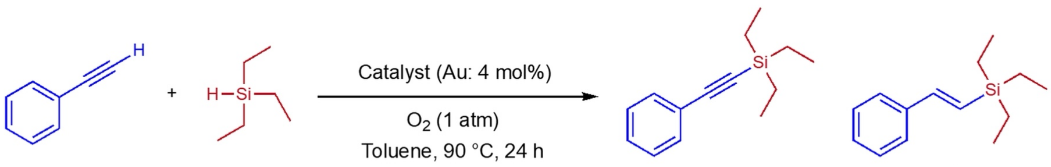

3 **4**

Catalyst	Yield of **3** (%)	Yield of **4** (%)
Au-**TOASiW9**	**61**	**Trace**
Au/OMS-2	81	**15**
Au/HAP	22	54

(c) Regiospecific alkynylation of 1-methylpiperidine using ethynylbenzene

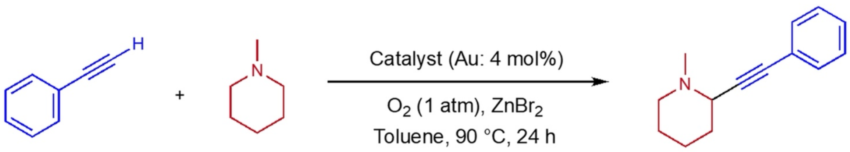

Catalyst	Yield (%)
Au-**TOASiW9**	**93**
Au/HAP	84

Supplementary Fig. 23 | Scope of catalytic reactions by Au-**TOASiW9** and conventional supported gold nanoparticle catalysts (e.g., Au/OMS-2 and Au/HAP). (a) oxidative dehydrogenation of *N*-alkyl-4-piperidone to the corresponding enaminone products; (b) cross-dehydrogenative coupling reaction of ethynylbenzene and triethylsilane; and (c) regiospecific alkynylation of 1-methylpiperidine using ethynylbenzene.

3. Supplementary Tables

Supplementary Table 1. Reported synthetic routes/preparation methods of gold nanoparticles with various stabilizing agents

Entry		Reducing agent	Stabilizing agent	Evaluation index for gold nanoparticles			Catalytic application
				Diameter (nm)	Concentration[a] (mM)	Surface[b]	
1	Faraday[3]	Phosphorus	Carbon sulfide	Ruby red solution	Not mentioned		–[c]
2	Turkevich[4]	Sodium citrate		18–20	0.3	B	Reaction of ferricyanide and thiosulphate
3	Frens[5]	Sodium citrate		16–150	0.5	B	
4	Brust & Schiffrin[6]	$NaBH_4$	Dodecane-thiol	1–3	11.3	**A**	Benzalacetone hydrogenation
5	Murphy[7]	$NaBH_4$	Citrate	3.5	0.3	B	Glycerol oxidation
6	Thomas & Kamat[8]	$NaBH_4$	TOAB[d]	~6	0.2	**A**	–[c]
7	Murphy[9]	$NaBH_4$	CTAB[e]	20–100	0.3	B	–[c]
8	Moores[10]	$Na^+C_{10}H_8^-$	Phosphinine[f]	5–20	13.5	**A**	–[c]
9	Tsukuda[11]	$NaBH_4$	PVP[g]	1.3, 9.5	0.9	B	Alcohol oxidation
10	Kobayashi[12]	$NaBH_4$	PI[h]	~1	2	**A**	Alcohol oxidation
11	Scrimin[13]	$NaBH_4$	Dioctyl-amine	1.9–8.9	1.0	B	Styrene oxidation
12	Santhanam[14]	Tannic acid		2–10	0.3	B	–[c]
13	Papaconstantinou[15]	**KSiW12**[i] + *hv*		13.1	0.1	B	–[c]
14	Cabuil[16]	$NaBH_4$	Citrate → **thiol-SiW10**[j]	4–12	2.0	B	–[c]
15	Maksimova[17]	CO	**Nb6**[k]	7.5–40	2.0	B	–[c]
16	Niu[18]	**HPW12**[l] + ascorbic acid		30–50	1.0	B	–[c]
17	Kulesza & Cox[19]	$NaBH_4$	Hexanethiol → **HPW12**[l]	4.4	0.5	B	Electrooxidation of cysteine
18	Nadjo[20]	**NaMo3S4**[m]		9.5	0.5	B	–[c]
19	Nadjo[21]	**HPMo12**[n] + electrolysis		~10	0.5	B	–[c]
20	Weinstock[22]	$NaBH_4$	Citrate → **KAlW11**[o]	13.8	0.3	B	CO oxidation
21	Weinstock[23]	$NaBH_4$	**KAlW11**[o]	~2	0.2	B	–[c]
22	Cronin[24]	Thermal	**TBAW12**[p]	20–100	1.3	B	–[c]
23	Polarz[25]	Thiol-**SiW11**[q] + *hv*		~50	0.3	B	Fischer esterification
24	Suzuki[26]	$NaBH_4$	**NaSiW9**[r]	~5	0.3	B	Oxidative dehydrogenation
**25**	**This work**	**$NaBH_4$**	**TOASiW9**[s]	**2.9**	**>5**	**A**	**Alcohol oxidation, oxidative dehydrogenation, cross-coupling, alkynylation**

[a]Concentration of initially added gold precursors in final solution used. [b]A = hydrophobic surface, B = hydrophilic surface. [c]Catalytic application was not mentioned in these reports. [d]Tetraoctylammonium bromide. [e]Cetyltrimethylammonium bromide. [f]2,3,5,6-tetraphenylphos-phinine and 3,5-diphenylphosphinine. [g]Poly(*N*-vinyl-2-pyrrolidone). [h]Polymer containing polystyrene derivatives made of styrene, 4-vinylbenzyl glycidyl ether and methacrylic acid. [i]$K_4[SiW_{12}O_{40}]$. [j]$TBA_4[SiW_{10}O_{36}(HSC_6H_3Si)_2O]$ (TBA = tetrabutylammonium). [k]$K_7H[Nb_6O_{17}]$. [l]$H_3[PW_{12}O_{40}]$. [m]$Na_2[Mo_3(\mu_3\text{-}S)(\mu\text{-}S)_3(Hnta)_3]$ (Hnta = nitrilotriacetic acid). [n]$H_3[H_4PMo_{12}O_{40}$ [o]$K_9[AlW_{11}O_{39}]$. [p]$TBA_4[H_4W_{12}O_{40}]$. [q]$H_4[SiW_{11}O_{39}(SiC_nH_{2n}SH)_2]$ (n = 4–10). [r]$Na_{10}[SiW_9O_{34}]$. [s]$TOA_xNa_{10-x}[SiW_9O_{34}]$ (TOA = tetraoctylammonium).

Supplementary Table 2. Results of elemental analysis for investigating percentages of transferred **SiW9** into toluene phase (i.e., **TOASiW9**) from initial **SiW9** in water phase (i.e., **NaSiW9**) under different adding amounts of TOAB and reductant ($NaBH_4$) with respect to $HAuCl_4$.

Entry	TOAB (equivalent to $HAuCl_4$)	$NaBH_4$ (equivalent to $HAuCl_4$)	**TOASiW9**/initial **SiW9** (mol%)
1	2	10	<1
2	4	10	<1
3	6	10	<1
4	8	10	2
5	10	10	6
6	10	8	10
7	10	6	24
**8**	**10**	**4**	**~40 (maximum transfer)**
9	10	2	40 (precipitation of gold)

Supplementary Table 3 | Selective aerobic oxidation of benzyl alcohol (1a) to benzaldehyde (2a) using different catalysts.[a]

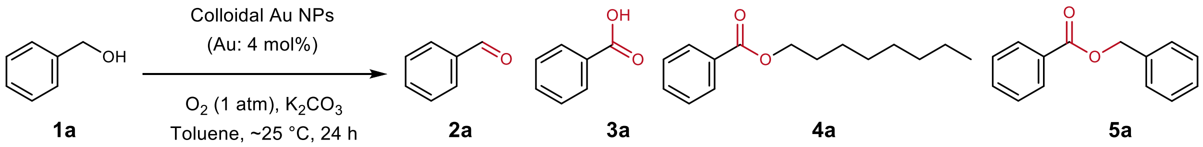

Entry	Catalyst	Conversion of **1a** (%)	Yield (%)			
			**2a**	**3a**	**4a**[b]	**5a**
1	Au-**TOASiW9**	84	75	n.d.	4	n.d.
2	Au-**TOASiW9**[c]	>99	92	n.d.	5	n.d.
3	Au-**TOASiW10**	23	17	n.d.	2	n.d.
4	Au-**TOASiW11**	26	24	n.d.	2	n.d.
5	Au-**TOASiW12**	6	4	n.d.	<1	n.d.
6	Au-TOAB	5	3	n.d.	<1	n.d.
7	Au-dodecanethiol	1	<1	n.d.	<1	n.d.
8	Au-dodecanethiol[c]	1	<1	n.d.	<1	n.d.
9	**TOASiW9**	<1	<1	n.d.	<1	n.d.
10	Au-**TOASiW9** (Ar 1 atm)[d]	<1	<1	n.d.	<1	n.d.

[a] Reaction conditions: **1a** (0.25 mmol), 3 mL toluene solution of colloidal gold nanoparticles (Au: 4 mol%), K_2CO_3 (0.5 mmol), room temperature (~25 °C), O_2 (1 atm), 24 h. [b] Formed by reaction of **3a** with octylhalide, decomposition product of TOAB. [c] Cs_2CO_3 (0.5 mmol) was used instead of K_2CO_3 (0.5 mmol). [d] Freeze-pump-thaw cycles were carried out and the reactor was connected to a balloon filled with an Ar gas.

4. Supplementary References

1 Yamaguchi, K., Wang, Y., Oishi, T., Kuroda, Y. & Mizuno, N. Heterogeneously catalyzed aerobic cross-dehydrogenative coupling of terminal alkynes and monohydrosilanes by gold supported on OMS-2. *Angew. Chem. Int. Ed.* **52**, 5627−5630 (2013).

2 Yatabe, T. & Yamaguchi, K. Regiospecific α-methylene functionalization of tertiary amines with alkynes via Au-catalysed concerted one-proton/two-electron transfer to O_2. *Nat. Commun.* **13**, 6505 (2022).

3 Faraday, M. The Bakerian lecture: –experimental relations of gold (and other metals) to light. *Philos. Trans. R. Soc. London* **147**, 145−181 (1857).

4 Turkevich, J., Stevenson, P. & Hiller, J. A study of the nucleation and growth processes in the synthesis of colloidal gold. *Discuss. Faraday Soc.* **11**, 55−75 (1951).

5 Frens, G. Controlled nucleation for the regulation of the particle size in monodisperse gold suspensions. *Nat. Phys. Sci.* **241**, 20−22 (1973).

6 Brust, M., Walker, M., Bethell, D., Schiffrin, D. & Whyman, R. Synthesis of thiol-derivatised gold nanoparticles in a two-phase liquid–liquid system. *J. Chem. Soc. Chem. Commun.* 801−802 (1994).

7 Jana, N. R., Gearheart, L. & Murphy, C. J. Seeding growth for size control of 5−40 nm diameter gold nanoparticles. *Langmuir* **17**, 6782−6786 (2001).

8 Thomas, K. G., Zajicek, J. & Kamat, P. V. Surface binding properties of tetraoctylammonium bromide-capped gold nanoparticles. *Langmuir* **18**, 3722−3727 (2002).

9 Sau, T. K. & Murphy, C. J. Seeded high yield synthesis of short Au nanorods in aqueous solution. *Langmuir* **20**, 6414−6420 (2004).

10 Moores, A., Goettmann, F., Sanchez, C. & Le Floch, P. Phosphinine stabilised gold nanoparticles; synthesis and immobilization on mesoporous materials. *Chem. Commun.* 2842−2843 (2004).

11 Tsunoyama, H., Sakurai, H., Negishi, Y. & Tsukuda, T. Size-specific catalytic activity of polymer-stabilized gold nanoclusters for aerobic alcohol oxidation in water. *J. Am. Chem. Soc.* **127**, 9374−9375 (2005).

12 Miyamura, H., Matsubara, R., Miyazaki, Y. & Kobayashi, S. Aerobic oxidation of alcohols at room temperature and atmospheric conditions catalyzed by reusable gold nanoclusters stabilized by the benzene rings of polystyrene derivatives. *Angew. Chem. Int. Ed.* **46**, 4151−4154 (2007).

13 Manea, F., Bindoli, C., Polizzi, S., Lay, L. & Scrimin, P. Expeditious synthesis of water-soluble, monolayer-protected gold nanoparticles of controlled size and monolayer composition. *Langmuir* **24**, 4120−4124 (2008).

14 Sivaraman, S. K., Kumar, S. & Santhanam, V. Room-temperature synthesis of gold nanoparticles –size-control by slow addition. *Gold. Bull.* **43**, 275−286 (2010).

15 Troupis, A., Hiskia, A. & Papaconstantinou, E. Synthesis of metal nanoparticles by using polyoxometalates as photocatalysts and stabilizers. *Angew. Chem. Int. Ed.* **41**, 1911−1914 (2002).

16 Mayer, C. R., Neveu, S. & Cabuil, V. A nanoscale hybrid system based on gold nanoparticles and heteropolyanions. *Angew. Chem. Int. Ed.* **41**, 501−503 (2002).

17 Maksimova, G. M., Chuvilin, A. L., Moroz, E. M., Likholobov, V. A. & Matveev, K. I. Preparation of colloidal solutions of noble metals stabilized by polyoxometalates and supported catalysts based on these solutions. *Kinet. Catal.* **45**, 870−878 (2004).

18 Yuan, J., Chen, Y., Han, D., Zhang, Y., Shen, Y., Wang, Z. & Niu, L. Synthesis of highly faceted multiply twinned gold nanocrystals stabilized by polyoxometalates. *Nanotechnology* **17**, 4689−4694 (2006).

19 Ernst, A. Z., Sun, L., Wiaderek, K., Kolary, A., Zoladek, S., Kulesza, P. J. & Cox, J. A. Synthesis of polyoxometalate-protected gold nanoparticles by a ligand-exchange method: application to the electrocatalytic reduction of bromate. *Electroanalysis* **19**, 2103−2109 (2007).

20 Keita, B., Biboum, R. N., Mbomekallé, I. M., Floquet, S., Simonnet-Jégat, C., Miserque, F., Berthet, P. & Nadjo, L. One-step synthesis and stabilization of gold nanoparticles in water with the simple oxothiometalate $Na_2[Mo_3(\mu_3\text{-}S)(\mu\text{-}S)_3(Hnta)_3]$. *J. Mater. Chem.* **18**, 3196−3199 (2008).

21 Zhang, G., Keita, B., Biboum, R. N., Miserque, F., Berthet, P., Dolbecq, A., Mialane, P., Catala, L. & Nadjo, L. Synthesis of various crystalline gold nanostructures in water: The polyoxometalate $\beta\text{-}[H_4PMo_{12}O_{40}]^{3-}$ as the reducing and stabilizing agent. *J. Mater. Chem.* **19**, 8639−8644 (2009).

22 Wang, Y., Neyman, A., Arkhangelsky, E., Gitis, V., Meshi, L. & Weinstock, I. A. Self-assembly and structure of directly imaged inorganic-anion monolayers on a gold nanoparticle. *J. Am. Chem. Soc.* **131**, 17412−17422 (2009).

23 Wang, Y., Raula, M., Wang, Y., Zeiri, O., Chakraborty, S., Gan-Or, G., Gadot, E. & Weinstock, I. A. Polyoxometalate-engineered building blocks with gold cores for the self-assembly of responsive water-soluble nanostructures. *Angew. Chem. Int. Ed.* **56**, 7083−7087 (2017).

24 Martín, S., Takashima, Y., Lin, C. G., Song, Y. F., Miras, H. N. & Cronin, L. Integrated synthesis of gold nanoparticles coated with polyoxometalate clusters. *Inorg. Chem.* **58**, 4110−4116 (2019).

25 Sutter, S., Trepka, B., Siroky, S., Hagedorn, K., Theiβ, S., Baum, P. & Polarz, S. Light-triggered boost of activity of catalytic bola-type surfactants by a plasmonic metal−support interaction effect. *ACS Appl. Mater. Interfaces* **11**, 15936−15944 (2019).

26 Xia, K., Yatabe, T., Yonesato, K., Yabe, T., Kikkawa, S., Yamazoe, S., Nakata, A., Yamaguchi, K. & Suzuki, K. Supported anionic gold nanoparticle catalysts modified using highly negatively charged multivacant polyoxometalates. *Angew. Chem. Int. Ed.* **61**, e202205873 (2022).
